# Reduced graphene oxide-incorporated gelatin-oxidized polymannose scaffold-based 3D Lung cancer model for mimicking the tumor microenvironment

**DOI:** 10.3389/fonc.2026.1955340

**Published:** 2026-09-24

**Authors:** Hema Priya Manivannan, Vishnu Priya Veeraraghavan, K. P. Ameya, Durairaj Sekar, Arul Prakash Francis

**Affiliations:** 1 Centre of Molecular Medicine and Diagnostics (COMManD), Department of Biochemistry, Saveetha Dental College and Hospitals, Saveetha Institute of Medical and Technical Sciences, Saveetha University, Chennai, India; 2 RNA Biology Lab, Saveetha Dental College and Hospital, Saveetha Institute of Medical and Technical Sciences, Saveetha University, Chennai, India

**Keywords:** 3D culture, gene expression, hypoxia, lung cancer, scaffold, tumor microenvironment

## Abstract

**Objective:**

The tumor microenvironment (TME) plays a critical role in regulating cancer progression, metastasis, and therapeutic response. Conventional two-dimensional (2D) culture systems fail to accurately recapitulate the complex native TME, highlighting the need for physiologically relevant three-dimensional (3D) *in vitro* models. In our previous work we have developed gelatin–oxidized polymannose–reduced graphene oxide (Gel: OPM:rGO) scaffold as a 3D lung cancer culture model. In this study, we aimed to assess its potential to support TME-associated responses compared with conventional 2D and Matrigel-based 3D culture systems.

**Materials and methods:**

A549 cells cultured on the Gel: OPM:rGO scaffold were analyzed for the secretion of growth factors and inflammatory cytokines by ELISA, the expression of TME-associated and signaling pathway-related genes by qRT-PCR, and TME-associated proteins by Western blotting. Metabolic profiling was performed by measuring glucose consumption and lactate production and the results were compared with 2D and Matrigel culture systems.

**Results:**

Gene expression analysis revealed the upregulation of TME-associated genes, including the hypoxia marker VEGF-A, the extracellular matrix (ECM) remodeling marker MMP9, the epithelial–mesenchymal transition (EMT) marker VIM, the oncogenic marker ALK, and the growth factor receptor EGFR. Western blot analysis showed increased expression of TME-related proteins in the Gel: OPM:rGO scaffold compared with conventional 2D culture. Major signaling pathways, including mTOR, PI3K/AKT, Wnt, c-Myc, STAT3, and TNF-α, were also modulated, indicating physiologically relevant cellular responses. Metabolic profiling in Gel: OPM:rGO culture supernatant demonstrated enhanced glucose consumption and lactate production, consistent with the Warburg effect and tumor-associated metabolic reprogramming. Overall, gene and protein expression profiles in the Gel: OPM:rGO scaffold exhibited trends similar to those observed in Matrigel-based 3D culture.

**Conclusion:**

Collectively, these findings demonstrate that the Gel: OPM:rGO scaffold is a robust and physiologically relevant 3D lung cancer model. As a potential 3D culture platform that complements Matrigel-based models, it provides a valuable *in vitro* platform for investigating tumor-associated cellular and molecular responses, cancer progression, and preclinical anticancer drug evaluation.

## Introduction

Lung cancer remains the most common cause of cancer-related deaths globally, with a projected 67% rise in incidence by 2040. Despite advances in detection and therapy, the 5-year survival rate remains low at around 10–20%, underscoring its aggressive nature and the ongoing public health challenge (1). Non-small cell lung cancer (NSCLC) constitutes approximately 85% of all lung cancer diagnoses (2). Despite advances in drug development, progress in developing anticancer drugs has been limited by the absence of tumor models that accurately replicate the complexity of human disease (3–5). The clinical approval rate for new lung cancer treatments remains unsatisfactory. A key reason for this persistent challenge is the lack of preclinical models that can accurately replicate the complex nature of the lung tumor microenvironment (TME) (6–11). TME is highly complex and functionally heterogeneous, with its spatial organization, biochemical gradients, and mechanical properties varying significantly even within a single tumor (12, 13).

Traditional two-dimensional (2D) cell cultures have long been the standard in cancer research and drug screening, but they lack the complexity and three-dimensionality of *in vivo* tumors, limiting their predictive power for drug screening and the study of cancer biology. These 2D models fail to accurately replicate cell-cell and cell-ECM interactions and the spatial organization that is pivotal to the authentic TME. These inadequacies can lead to misleading results regarding drug responses, resistance mechanisms, and tumor biology, ultimately contributing to the high failure rates of new lung cancer drugs in clinical trials (14–16). In contrast, three-dimensional (3D) cell culture systems address the limitations of 2D culture by providing a more realistic and physiologically relevant *in vitro* platform that better mimics the *in vivo* TME, making them valuable tools for cancer research and drug discovery (17, 18). 3D culture recapitulates key features of lung cancer, such as hypoxic and nutrient gradients, cellular heterogeneity, and dynamic cell–cell and cell-extracellular matrix (ECM) interactions. These platforms allow more accurate investigation of tumor progression, metastasis, drug penetration, and resistance compared to 2D cultures (19–21).

To address the limitations of conventional culture models, we employed our previously reported reduced graphene oxide-incorporated gelatin-polymannose (Gel: OPM:rGO) scaffold as a 3D lung cancer model. The scaffold fabrication, physicochemical characterization, and biocompatibility have been described in our previous study (22). Based on these findings, the optimized 3:5 Gel: OPM and Gel: OPM:rGO scaffold formulations, which demonstrated the most favorable physicochemical properties and biocompatibility, were selected for the present study. A549 lung cancer cells were cultured within the optimized Gel: OPM:rGO scaffold to evaluate its ability to recreate a physiologically relevant lung TME. The performance of the Gel: OPM:rGO scaffold was systematically compared with 2D culture, Matrigel-based 3D culture, and the corresponding Gel: OPM scaffold. Specifically, we evaluated the scaffold’s ability to support tumor-like cellular architecture, cell–matrix interactions, and selected cellular and molecular features associated with the lung TME. This work demonstrates the potential of the Gel: OPM:rGO scaffold as a physiologically relevant *in vitro* platform for 3D lung cancer modelling using A549 cells.

## Materials and methods

Oxidized Polymannose (OPM) (Mol. wt. 18 kDa) was prepared from polymannose (Mol. wt. 53 kDa) obtained using the polycondensation of mannose units, as reported earlier in our previous study (23). Sodium metaperiodate, Isopropanol, Bovine serum albumin (BSA), sodium bicarbonate, Sodium dodecyl sulfate (SDS), Agarose, reduced graphene oxide (rGO), and Bicinchoninic acid (BCA) were purchased from SRL Chemicals India. Dulbecco’s Modified Eagle Medium (DMEM), Fetal Bovine Serum (FBS), Trypsin-EDTA, Alamar Blue, and Ethidium Bromide were purchased from Himedia. Gelatin (Type A gelatin from porcine skin), Paraformaldehyde, DAPI, 2′,7′-dichlorodihydrofluorescein diacetate (DCFDA), and dimethyl sulfoxide (DMSO) were procured from Sigma-Aldrich. Rhodamine 123 was purchased from Thermofisher. Penicillin–streptomycin antibiotic was procured from Gibco. Dialysis tubing (molecular weight cut-off (MWCO) 6–8 kDa) was purchased from Spectrum Chemicals. Trizol reagent, RT mastermix, and qPCR premixes were procured from Takara. The primers were synthesized by Eurofins. Matrigel basement membrane matrix and cell recovery solution were purchased from Corning. EGF, VEGF, bFGF, TGF-β1, IL-8, IL-6, and TNF-α ELISA Kits were procured from Abbkine. Primary antibodies - Anti-EGFR, Anti-ALK, anti-MMP9, anti-VEGF A, anti- GAPDH, and anti- VEGF-A antibody were purchased from Cell Signaling Technology. All primary antibodies used in this study were rabbit monoclonal antibodies. Anti-rabbit IgG, HRP-linked Antibody was purchased from Cell Signaling Technology. Human alveolar adenocarcinoma cells (A549 cell line) were obtained from NCCS, India.

### Cell growth and maintenance

Human lung adenocarcinoma A549 cells were maintained in a T25 cell culture flask with Dulbecco’s Modified Eagle Medium (DMEM) supplemented with 10% FBS and 1% Penicillin/streptomycin in a humidified chamber at 37 °C and 5% CO_2_. 0.25% Trypsin-EDTA solution was employed for cell detachment during passaging.

### Scaffold fabrication

The 3:5 Gel: OPM:rGO and 3:5 Gel: OPM scaffolds used in this study were fabricated according to our previously published protocol (22). The detailed fabrication, physicochemical characterization and biocompatibility of these scaffolds were reported in the same previous study (22). For ease of reference, a summary of the key scaffold properties, including swelling and degradation behavior, porosity, mechanical properties, and biocompatibility, is provided in the **Supplementary File**.

### 3D scaffold culture

Prior to cell seeding, the Gel: OPM and Gel: OPM:rGO scaffolds were sterilized under UV light for 1 h and pre-equilibrated with 1 mL of complete culture medium at 37 °C in a humidified incubator with 5% CO_2_ for 2 h. Subsequently, A549 cells (7.5 × 10^4^ cells per scaffold) were seeded onto each scaffold and cultured at 37 °C in a humidified atmosphere containing 5% CO_2_. The culture medium was replaced every 48 h throughout the culture period.

### 3D Matrigel culture

Corning Matrigel basement membrane matrix is a widely used and reliable ECM product for cell culture applications. It is a solubilized basement membrane extract derived from Engelbreth-Holm-Swarm (EHS) mouse sarcoma, which is naturally rich in ECM proteins and growth factors. Matrigel was stored at −20 °C and thawed overnight on ice at 4 °C before use. In this study, the “on-top” culture method was employed, and the procedure recommended by Corning was followed. Briefly, 200 μL of Matrigel was added to each well of a pre-chilled 24-well plate using cooled pipette tips. The Matrigel was evenly spread and incubated at 37 °C for 30 minutes to allow solidification. Approximately 7.5x10^4^ A549 cells were seeded onto the solidified Matrigel and incubated at 37 °C for 30 minutes to facilitate cell attachment. After this, a chilled Matrigel-medium mixture was gently overlaid onto the cells. Cultures were maintained for 7 days, with medium changed every 2 days.

### Quantification of secreted cytokines and growth factors proteins

Culture supernatants from scaffolds and Matrigel systems were collected on days 4 and 7, while the supernatants from 2D cultures maintained at 6 well plates were collected on day 4. The levels of secreted growth factors (VEGF, bFGF, TGF-β1, and EGF) and inflammatory cytokines (IL-6, IL-8, and TNF-α) were measured using commercially available enzyme-linked immunosorbent assay (ELISA) kits (Abbkine), following the manufacturer’s instructions. Absorbance was measured at 450 nm using a microplate reader. To account for differences in cell abundance among the experimental groups, the ELISA results were normalized to the corresponding total cellular protein content.

### Comparative expression analysis of key TME and signaling pathway genes

The expression levels of key TME-associated genes from scaffolds and Matrigel were analyzed on Days 4 and 7 using quantitative reverse transcription polymerase chain reaction (qRT-PCR). In 2D culture, the RNA was extracted on day 4. The genes selected for expression analysis included the hypoxia marker (VEGF-A), the ECM degradation marker (MMP9), the epithelial–mesenchymal transition (EMT) markers (VIM and ALK), and the growth factor receptor EGFR. Similarly, the expression levels of major signaling pathway-related genes in NSCLC, including mTOR, AKT, PI3K, WNT, STAT3, TNF-α, c-MYC, and p53, were also analyzed to identify alterations in key cellular signaling pathways under 2D and 3D conditions.

Total RNA was extracted from 2D cultures by trypsinization, followed by resuspension in TRIzol reagent (Takara). For 3D scaffold cultures, cells were lysed directly on the scaffolds using TRIzol. In the case of Matrigel cultures, cells were first recovered using Cell Recovery Solution, then resuspended in TRIzol for RNA isolation. RNA samples collected on Days 4 and 7 were quantified using a NanoDrop spectrophotometer (Thermo Fisher Scientific). Subsequently, 1 μg of total RNA was reverse-transcribed into cDNA. qRT-PCR was carried out using the TB Green Advantage qPCR Premix (Takara). The reaction mixture contained 12.5 μL of 2× SYBR Green Master Mix, 300 nM of forward and reverse primers, and 1 μL of cDNA (100 ng/μL). Amplification was performed using a CFX96 real-time PCR detection system (Bio-Rad). Primer sequences used for each target gene are listed in **Supplementary Tables S1** and **S2**. Gene expression levels were normalized to GAPDH, and relative quantification was calculated using the comparative (ΔΔCt) method. Data are presented as fold changes (2^–ΔΔCt), with expression levels in 2D cultures serving as the baseline control for comparison against 3D scaffold and Matrigel cultures (24).

### Western blotting

Western blotting was performed to analyze the expression of key TME-related proteins. Total cellular protein was extracted from 2D cultures on day 2 and from 3D Matrigel cultures, Gel: OPM:rGO scaffolds, and Gel: OPM scaffolds at day 7 using radioimmunoprecipitation assay (RIPA) buffer. The lysates were clarified by centrifugation, and protein concentrations were determined using the Lowry method. Equal amounts of protein were resolved by sodium dodecyl sulfate–polyacrylamide gel electrophoresis (SDS-PAGE) and subsequently transferred onto nitrocellulose membranes. The membranes were blocked with 5% skim milk prepared in Tris-buffered saline containing 0.1% Tween-20 (TBS-T) to prevent nonspecific binding. Following blocking, the membranes were incubated overnight at 4 °C with primary antibodies against ALK, EGFR, MMP-9, VEGF-A, vimentin, and GAPDH (1:1000; Cell Signaling Technology). After incubation, the membranes were washed three times with TBS-T and then incubated with horseradish peroxidase (HRP)–conjugated secondary antibody (1:2500; Cell Signaling Technology) for 1 h at room temperature. The membranes were washed thoroughly and developed using 3,3′-diaminobenzidine (DAB) substrate (Thermo Fisher Scientific, USA) under dark conditions. Protein bands were visualized, imaged, and quantified using ImageJ software. Protein band intensities were normalized to GAPDH and expressed relative to the 2D culture condition.

### Glucose–lactate metabolism profiling

Glucose–lactate metabolism was assessed under both 2D and 3D culture conditions. Culture supernatants were collected at various time points, and glucose levels were measured using the Glucose Oxidase–Peroxidase (GOD-POD) colorimetric assay (Diatek). Lactate concentration was determined following the procedure described by Schmiedeknecht et al. (25, 26). The culture medium was not replaced during the 72 h experimental period in any culture condition to allow continuous assessment of metabolic changes.

### Statistical analysis

Experiments were performed using three independent biological replicates (*n* = 3), except for Western blot experiments, which were performed using two independent biological replicates (*n* = 2). Data are presented as mean ± standard error of the mean (SEM). Statistical comparisons were performed using one-way analysis of variance (ANOVA), followed by Dunnett’s multiple-comparison test with the 2D group used as the reference control. For RT-qPCR analysis, statistical comparisons were performed using the corresponding ΔCt values obtained from individual biological replicates. Relative gene expression was calculated using the 2^−ΔΔCt method and is presented as fold change. A *p*-value of less than 0.05 was considered statistically significant.

## Results

### Quantification of secreted cytokines, growth factors, and ECM proteins

The levels of growth factors (VEGF, bFGF, TGF-β1, and EGF) and inflammatory cytokines (IL-6, IL-8, and TNF-α) in the culture supernatants collected on days 4 and 7 were quantified using ELISA and normalized to the corresponding total cellular protein content (**Figure 1**). On day 7, growth factor secretion by cells cultured on the Gel: OPM:rGO scaffold showed similar overall trends to those observed in the 3D Matrigel culture. Similarly, inflammatory cytokine secretion was elevated in the Gel: OPM:rGO scaffold on day 7. Although slightly lower than the levels observed in 3D Matrigel, the cytokine secretion was significantly higher than in the 2D and Gel: OPM cultures. In contrast, cells maintained under 2D conditions secreted considerably lower levels of both growth factors and cytokines. Overall, both growth factors and inflammatory cytokines showed increased expression on day 7 compared to day 4 across all 3D culture systems.

**Figure 1:**
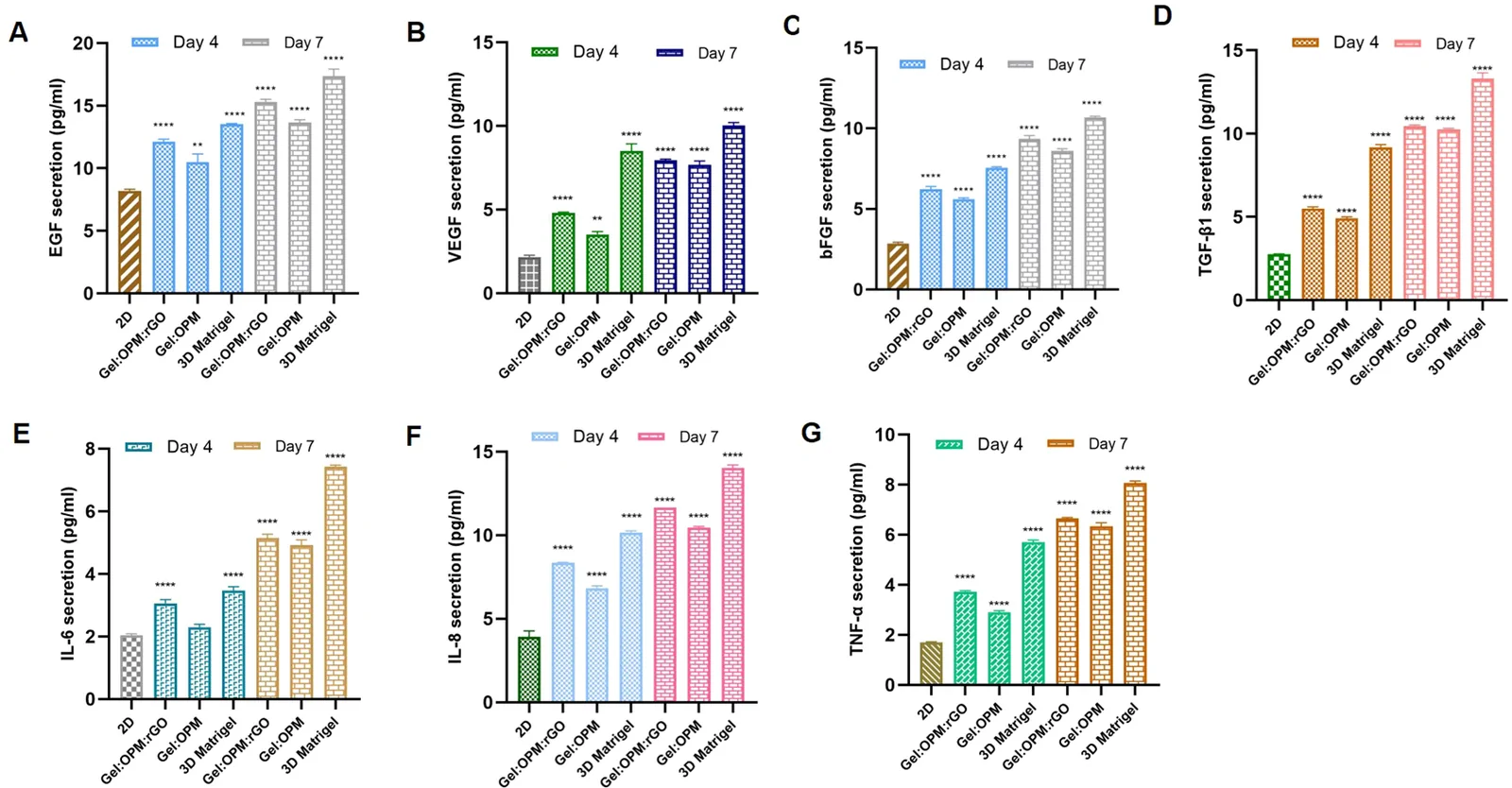
Comparison of growth factor and inflammatory cytokines expression profiles of A549 cells cultured *in vitro* for day 4 and day 7 **(A)** EGF, **(B)** VEGF, **(C)** bFGF, **(D)**TGF-β1**(E)** IL-6, **(F)** IL-8 **(G)** TNF-α -secretion by A549 cells cultured on 2D, Gel: OPM;rGO, Gel: OPM and 3D Matrigel as determined by ELISA and normalized to the corresponding total cellular protein content. Results are expressed as mean ± SE., and **indicates p < 0.01, ***indicates p < 0.001, and ****indicates p < 0.0001 (n = 3).

The increased levels of VEGF and bFGF observed in the 3D culture systems may be associated with angiogenic signaling, while the elevated levels of TGF-β1 and EGF may reflect alterations in signaling pathways related to tumor cell behavior. Similarly, the increased secretion of IL-6, IL-8, and TNF-α indicates differences in the inflammatory cytokine profile among the culture conditions. These factors have previously been associated with processes involved in tumor progression, including angiogenesis, cell survival, invasion, and inflammatory signaling (27–33). Overall, these findings demonstrate that the Gel: OPM:rGO scaffold provides a supportive microenvironment for cancer cells by stimulating both angiogenic growth factor release and inflammatory cytokine signaling, with several observed patterns resembling those observed in Matrigel-based culture. Compared with 2D cultures, these expressions in 3D culture were statistically significant.

### Comparative analysis of TME-related gene expression

The relative expression of genes regulating the TME, including hypoxia (VEGF-A), ECM degradation (MMP9), EMT markers (VIM and ALK), and growth factor receptor marker (EGFR), was analyzed using qRT-PCR on days 4 and 7 in A549 cells cultured in Gel: OPM:rGO, Gel: OPM, and3D Matrigel. While the 2D monolayers the samples were collected on day 4 (**Figure 2**). VEGF-A expression on day 4 showed differential regulation among the 3D culture systems. Expression was maintained at a level comparable to the 2D culture (day 4 Sample) in Gel: OPM:rGO (1.02-fold) and 3D Matrigel (1.1-fold), whereas Gel: OPM showed decreased expression (0.39-fold). By day 7, VEGF-A expression increased to 1.8-fold in Gel: OPM:rGO, 1.82-fold in Gel: OPM, and 3.45-fold in Matrigel relative to the 2D culture, suggesting enhanced VEGF-A expression in the 3D culture systems at this time point. MMP9, a gene associated with ECM remodeling, showed increased expression at day 4. MMP9 expression increased in Gel: OPM:rGO (2.14-fold) and Matrigel (2.19-fold), whereas decreased expression was observed in Gel: OPM (0.68-fold). By day 7, MMP9 expression decreased in Gel: OPM:rGO (0.65-fold) and Gel: OPM (0.23-fold), whereas increased expression was maintained in Matrigel (1.73-fold). EGFR expression was markedly upregulated throughout the culture period in Gel: OPM:rGO, reaching 5.7-fold on day 7, while a 5.3-fold increase was observed in Matrigel, showing a similar overall expression levels between the two 3D culture conditions. Similarly, the EMT marker VIM was significantly upregulated in Gel: OPM:rGO, showing 4.2-fold and 4.65-fold increases on days 4 and 7, respectively, while Matrigel exhibited increases of 5.6-fold and 5.59-fold. Gel: OPM also showed increased VIM expression, although at lower levels with 2.12-fold and 3.01-fold increases on days 4 and 7, respectively. ALK expression was likewise elevated in the 3D culture systems. Gel: OPM:rGO exhibited a 1.04-fold increase on day 4, followed by decreased expression (0.66-fold) on day 7. Similarly, Matrigel showed decreased expression on day 4 (0.89-fold) and a slight increase on day 7 (1.06-fold), whereas Gel: OPM displayed increased expression at both time points (1.11-fold and 1.16-fold on days 4 and 7, respectively). Overall, the expression profiles of key TME-related genes, including VEGF-A, MMP9, EGFR, VIM, and ALK, in the Gel: OPM:rGO scaffold displayed a broadly similar expression pattern to that observed in the Matrigel-based 3D culture, particularly on day 7. In contrast, gene expression levels in Gel: OPM were consistently lower, suggesting a contribution of rGO to the observed molecular responses.

**Figure 2:**
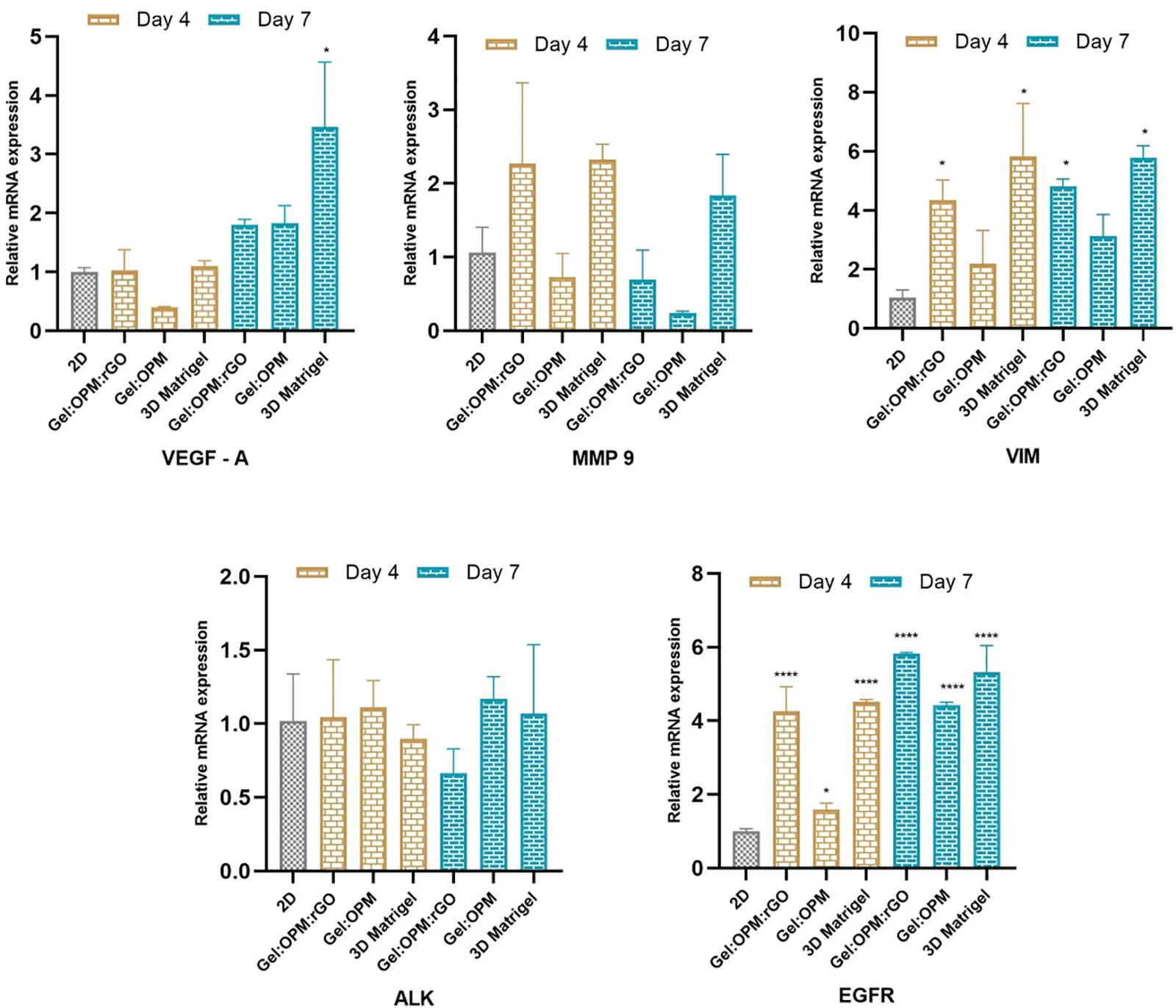
qRT-PCR of TME - related gene expression in 2D, 3D Gel:OPM:rGO, Gel:OPM, and 3D Matrigel. Results are expressed as mean ± SE, and * indicates p>0.05, and **** indicates p<0.0001 compared to 2D culture (n=3).

These findings highlight the ability of the Gel: OPM:rGO scaffold to promote gene expression patterns associated with tumor progression, EMT, and ECM remodeling—closely resembling the 3D Matrigel system. This underscores the scaffold’s potential as a physiologically relevant 3D *in vitro* tumor model, superior to conventional 2D culture and Gel: OPM without rGO. Heatmaps of fold change in TME gene expression in scaffold and Matrigel cultures, compared to 2D, are shown in **Supplementary Figure S1**.

### Western blotting

The protein expression levels of key TME-associated proteins such as ALK, EGFR, MMP-9, VEGF-A, VIM, and GAPDH across all culture conditions were analyzed using Western blotting and are presented in **Figure 3**. GAPDH was used as an internal control for normalization and expressed relative to the 2D culture. Compared with the 2D culture, the Gel: OPM:rGO system exhibited increased protein expression. However, the 3D Matrigel culture showed the highest protein expression among all culture conditions. Although protein expression in the Gel: OPM scaffold was increased compared to the 2D culture, it remained lower than that observed in both Matrigel and Gel: OPM:rGO conditions. The Gel: OPM:rGO scaffold system showed protein expression levels that broadly resembled those observed in the Matrigel culture. Western blot data indicated that alterations in the culture microenvironment may predominantly influence TME-related proteins at the post-transcriptional level. Overall, protein expression was consistently higher in 3D cultures than in 2D cultures, indicating alterations in signaling pathways, ECM remodeling, hypoxia-associated signaling, and mesenchymal markers under 3D growth conditions.

**Figure 3:**
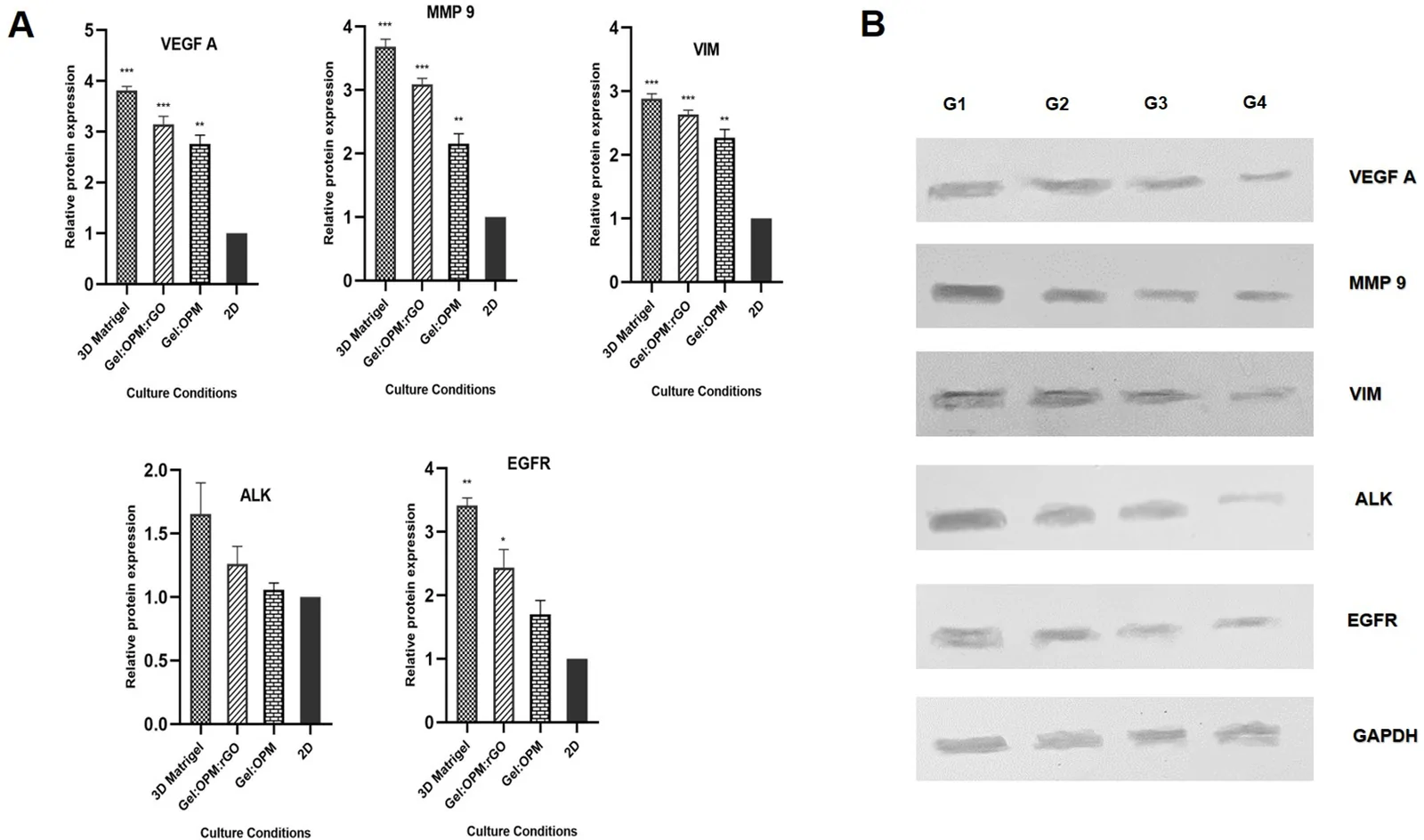
**(A)** Protein expression analysis of VEGF-A, MMP-9, VIM, ALK, and EGFR across different culture conditions using Western blotting. GAPDH was used as the loading control, and protein expression levels were normalized to GAPDH and expressed relative to the 2D culture condition. Results are expressed as mean ± SE (n=2), and *indicates p < 0.05, **indicates p < 0.01, ***indicates p < 0.001, and ****indicates p<0.0001 compared to 2D culture. **(B)** Western blot analysis showing the protein expression levels of VEGF-A, MMP-9, VIM, ALK, and EGFR in cells cultured under different conditions. GAPDH was used as the internal control. Culture groups include G1: 3D Matrigel, G2: Gel: OPM:rGO scaffold, G3: Gel: OPM scaffold, and G4: 2D monolayer culture.

### Comparative analysis of signaling pathway genes

In addition to TME-associated markers, major cancer signaling pathway markers such as mTOR, AKT, PI3K, Wnt, c-Myc, STAT3, TNF-α, and p53 were also analyzed by qRT-PCR (**Figure 4**). These pathways are central regulators of cell survival, proliferation, and tumor progression, where PI3K/AKT/mTOR signaling promotes growth and metabolic activity, Wnt and c-Myc drive uncontrolled proliferation, STAT3 and TNF-α mediate inflammation-driven survival, and p53 serves as a critical tumor suppressor controlling cell cycle arrest and apoptosis.

**Figure 4:**
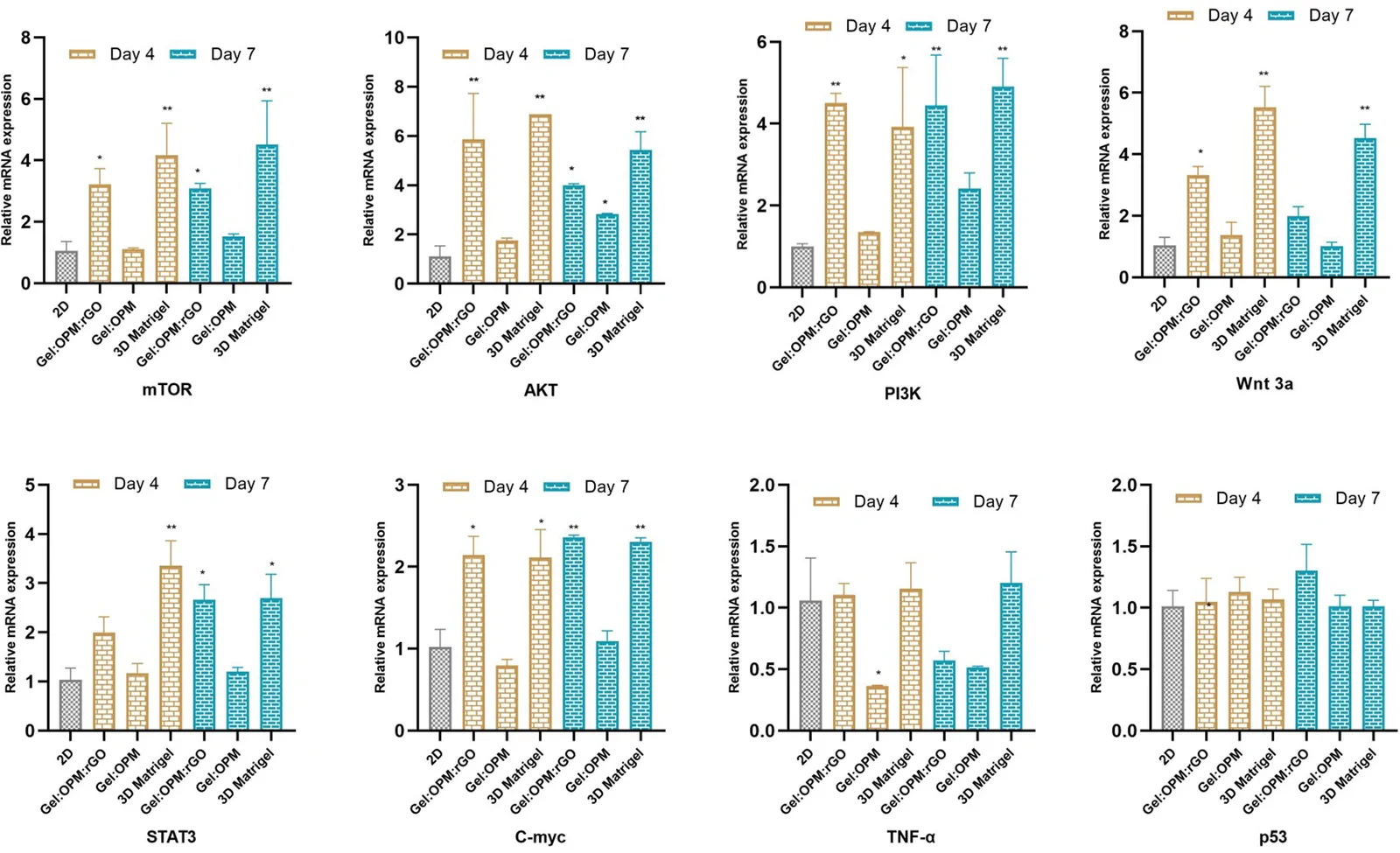
qRT-PCR of Signaling pathway gene expression in 2D, 3D Gel: OPM:rGO, Gel: OPM, and 3D Matrigel. Results are expressed as mean ± SE, and *indicates p < 0.05, and **indicates p < 0.01, compared to 2D culture (n=3).

qRT-PCR analysis revealed that 3D-cultured A549 cells exhibited altered signaling gene expression compared to 2D cultures. On Day 4, mTOR, AKT, and PI3K were upregulated in Gel: OPM:rGO scaffolds (3-, 5.3-, and 4.5-fold, respectively) and in Matrigel (4-, 6-, and 3.9-fold). By Day 7, increased expression persisted in Gel: OPM:rGO (3-, 3.6-, and 4.4-fold) and Matrigel (4-, 4.9-, and 4.9-fold), with Matrigel generally showing higher expression. This upregulation indicates altered expression of genes associated with survival and proliferation pathways potentially reflecting the influence of enhanced cell–matrix interactions and microenvironmental cues in Gel: OPM:rGO cultures, which provide a 3D microenvironment that captures key features observed in Matrigel culture. Although Gel: OPM cultured cells also showed upregulation, it was lower compared to Gel: OPM:rGO.

Wnt3a and c-Myc were also upregulated in 3D-cultured A549 cells compared to 2D cultures. On day 4, Wnt3a expression was increased by 3.2-fold in Gel: OPM:rGO and 5-fold in Matrigel, while c-Myc expression increased by 2-fold and 3-fold, respectively. By day 7, Wnt3a and c-Myc remained upregulated, with Gel: OPM:rGO showing 2-fold and 2.3-fold increases and Matrigel showing 4-fold and 2.8-fold increases, respectively. Overall, Gel: OPM:rGO induced Wnt3a and c-Myc expression, although to a slightly lower extent than Matrigel. These changes indicate altered expression of Wnt3a and c-Myc, which are associated with Wnt/β-catenin signaling and cellular processes such as proliferation, survival, and stemness.

STAT3 expression was increased in Gel: OPM:rGO and Matrigel on day 4, showing 2-fold and 3-fold expression, respectively, and increased to 2.6-fold in both culture systems by day 7, indicating sustained STAT3 upregulation. In contrast, TNF-α expression remained approximately unchanged (1-fold) on day 4 in both culture systems and decreased to 0.5-fold in Gel: OPM:rGO by day 7, while remaining approximately unchanged in Matrigel. These results suggest altered expression of genes associated with STAT3-mediated pro-survival and TNF-α-associated inflammatory signaling in 3D cultures, with TNF-α expression showing a reduction in Gel: OPM:rGO, which may reflect cellular adaptation to the 3D microenvironment. The expression of p53 remained approximately unchanged in the 3D scaffold cultures (fold change = 1) on both days 4 and 7, with similar expression levels observed in Matrigel. Heatmaps of fold change in signaling pathway gene expression in scaffold and Matrigel cultures, compared to 2D, are shown in **Supplementary Figure S2**.

### Glucose–lactate metabolism profiling

To evaluate the metabolic activity of A549 cells, glucose and lactate concentrations were measured in Gel: OPM:rGO and Gel: OPM scaffolds, as well as in Matrigel, and compared with 2D cultures (**Figure 5**). Glucose levels declined steadily in the culture medium of the scaffold and Matrigel groups over time, indicating a greater rate of glucose consumption than in 2D monolayer cultures. In contrast, lactate levels were significantly higher in the Gel: OPM:rGO, Gel: OPM scaffolds, and 3D Matrigel cultures relative to 2D cultures over time. These findings indicate differences in glucose and lactate metabolism among the different culture conditions. As these measurements were not normalized to cell abundance, the results should be interpreted as preliminary metabolic differences and do not allow a definitive conclusion regarding increased glycolytic activity in the 3D culture systems.

**Figure 5:**
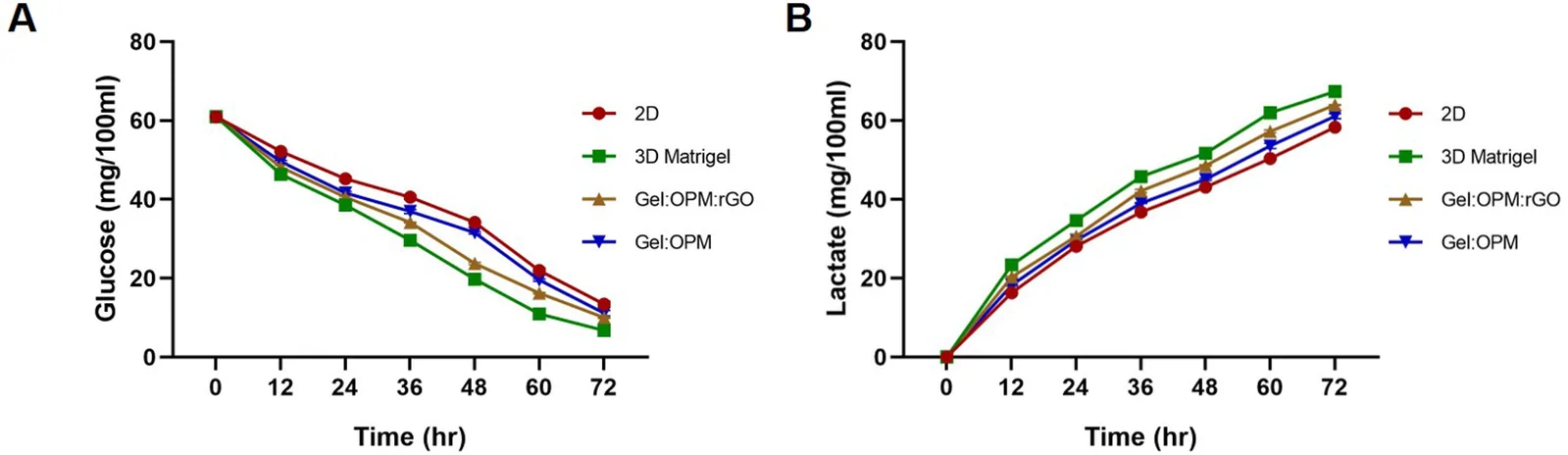
**(A)** Glucose and **(B)** Lactate metabolism in 2D and 3D cultures (n=3).

In cancer biology, the Warburg effect is a hallmark of cancer metabolism and refers to the tendency of most cancer cells to generate energy predominantly through glycolysis followed by lactic acid fermentation in the cytosol, rather than through mitochondrial oxidation of pyruvate as observed in normal cells. This metabolic reprogramming, also termed aerobic glycolysis, occurs even in the presence of sufficient oxygen. Cancer cells exhibit increased glucose uptake and preferentially convert it to lactate, leading to lactic acid accumulation. The resulting acidification of the extracellular microenvironment promotes tumor progression by enhancing malignant cell invasion and survival (34–38). Analysis of glucose and lactate levels revealed differences in the metabolic profiles of A549 cells cultured in 2D, Gel: OPM:rGO, Gel: OPM, and 3D Matrigel conditions. The observed decrease in glucose levels and increase in lactate levels in the 3D culture systems compared with 2D cultures, may be associated with altered cellular metabolism.

## Discussion

Lung cancer remains one of the leading causes of cancer-related deaths worldwide (39). Despite significant advances in treatment, clinical outcomes remain poor, reflecting the inadequacy of conventional preclinical models in predicting therapeutic responses (6). The tumor TME refers to the complex and dynamic network surrounding tumor cells, which plays a crucial role in cancer initiation, progression, and therapeutic resistance. The TME is an active participant in tumor evolution. Crosstalk between cancer cells and their surrounding microenvironment regulates invasion, EMT, metastasis, and therapeutic resistance in the TME (40–43). Recreating this complexity *in vitro* is essential for advancing the mechanistic understanding of cancer.

Scaffold-based 3D cancer cell culture systems have emerged as powerful platforms for studying tumor biology, as they provide a more physiologically relevant model that traditional 2D monolayer cultures. In these systems, natural or synthetic biomaterials are engineered into porous scaffolds, offering structural support and biochemical cues for cancer cell growth, migration, and interaction. Unlike 2D cultures, which lack spatial organization and fail to recapitulate the complexity of tumors, scaffold-based models allow cells to proliferate in three dimensions, forming aggregates and spheroids that resemble an *in vivo* tumor. Within 3D scaffolds, cancer cells exhibit altered morphology, polarity, and proliferation patterns, resembling the *in vivo* tumor tissue structure. The porous, heterogeneous nature of scaffolds facilitates gradients of oxygen, nutrients, and metabolites, inducing hypoxia and metabolic heterogeneity, both hallmarks of the TME (44–49).

In our previous study, the optimized Gel: OPM:rGO scaffold demonstrated superior physicochemical properties and biocompatibility compared with the Gel: OPM scaffold and exhibited biological responses that broadly resembled those observed in Matrigel. The scaffold supported sustained A549 cell growth for up to 7 days while providing a structurally supportive microenvironment that promoted cell–cell interactions and supported the expression of key molecular features associated with the lung TME, including the upregulation of hypoxia (HIF-1α), ECM remodeling (MMP2), Wnt/β-catenin signaling (β-catenin), and EMT marker CDH1. Building upon these findings, the present study further evaluated the ability of the optimized Gel: OPM:rGO scaffold to recapitulate additional biochemical characteristics of the lung TME in detail.

The ELISA analysis in our study provided critical insights into the impact of 3D culture systems on the secretion of tumor-associated growth factors and cytokines in the culture supernatants. Compared with conventional 2D cultures, cells grown in 3D scaffolds demonstrated significantly elevated levels of growth factors and inflammatory cytokines, indicating that the 3D microenvironment strongly influences cancer cell behavior. Leung et al. conducted a comparative ELISA to evaluate IL-8, bFGF, and VEGF secretion in 2D, 3D Matrigel, and scaffold-based cultures. Their findings showed that HepG2 and PLC hepatocellular carcinoma cells grown in a chitosan–alginate scaffold exhibited markedly increased secretion of these factors by day 9, with levels significantly higher than Matrigel-based cultures (27). Similarly, Chen et al. reported that MCF-7 cells cultured in a 3D collagen scaffold secreted higher levels of VEGF, bFGF, and IL-8 compared to 2D culture, particularly evident after 9 days of culture (50). In line with their findings, our ELISA results demonstrated that A549 cells cultured in 3D Gel: OPM:rGO scaffolds exhibited significantly elevated secretion of factors compared to 2D cultures, but lower levels than those observed in Matrigel. These findings suggest that the 3D culture environment influences the growth factor- and cytokine-associated signaling milieu. The observed differences may be associated with the enhanced cell–cell and cell–matrix interactions provided by the 3D microenvironment. However, because Matrigel contains ECM components and growth factors that may contribute to the levels detected in the culture supernatant, and because a cell-free Matrigel control was not included in the present study, the measured concentrations cannot be attributed exclusively to A549 cell secretion. Collectively, these findings suggest that the Gel: OPM:rGO scaffold supports tumor-associated molecular and secretory responses distinct from those observed in conventional 2D culture, while exhibiting certain features resembling those observed in Matrigel-based 3D culture.

To gain insights into the molecular mechanisms underlying cancer cell behavior within the 3D scaffold, we analyzed the expression of key TME-related genes, including EMT markers, growth factors, and ECM degradation markers, as well as major signaling pathway genes, using qRT-PCR. The upregulation of TME-related genes, ECM degradation markers, growth factors, and EMT markers in A549 cells cultured within the Gel: OPM:rGO scaffold suggests that our 3D scaffold effectively recreates a tumor-like microenvironment. The upregulation of VEGF-A observed in the Gel: OPM:rGO scaffold may reflect activation of hypoxia-associated pathways within the 3D microenvironment. However, hypoxia was not directly measured in the present study and VEGF-A expression can be influenced by multiple signaling pathways. Under hypoxic conditions, HIF-1α is stabilized and activated, acting as a key regulator of cellular adaptation to low oxygen tension and subsequently promoting VEGF-A expression to stimulate angiogenesis and support tumor progression (51, 52). Therefore, the observed increase in VEGF-A expression should be interpreted as being consistent with hypoxia-associated signaling rather than as direct evidence of hypoxia. The observed similarity in VEGF-A expression between the Gel: OPM:rGO scaffold and Matrigel further suggests that the scaffold may recapitulate certain hypoxia-associated features of the TME. Similarly, the elevated expression of MMP9 indicates active ECM remodeling, a process associated with cancer cell migration and invasion. The marked upregulation of EGFR indicates increased expression of a key receptor associated with growth factor-mediated signaling, cell proliferation, and survival. Furthermore, the increased expression of the EMT marker VIM, together with ALK, suggests molecular changes associated with tumor progression and metastatic potential. Collectively, these findings demonstrate that the Gel: OPM:rGO scaffold is a physiologically relevant 3D model for studying cancer progression, and drug responses.

Szot et al. reported that HIF-1α and VEGF-A were upregulated in MDA-MB-231 cells cultured in 3D collagen I hydrogels compared to 2D culture (53). Similarly, our study also demonstrated enhanced VEGF-A expression in 3D scaffold culture, which may be associated with hypoxia-related signaling. These results collectively suggest that 3D culture conditions alter gene expression and may provide a microenvironment that recapitulates certain features of the TME compared with conventional 2D monolayer cultures.

Compared with RT-qPCR results of TME-related genes, Western blot analysis revealed additional differences at the protein level, and the overall expression trends were not completely identical in Gel: OPM:rGO scaffolds. These differences may reflect regulatory mechanisms beyond transcription, potentially including post-transcriptional regulation. Importantly, differences in the time points used for qRT-PCR and Western blot analyses may also have contributed to the observed divergence between mRNA and protein expression patterns. While qRT-PCR was performed at days 4 and 7, Western blot analysis was conducted at day 7 for the 3D culture groups and day 2 for the 2D culture group. Therefore, temporal differences in gene and protein expression may have partially contributed to the observed non-identical expression trends. Accordingly, the divergence between mRNA and protein expression cannot be attributed exclusively to post-transcriptional regulation. Future studies evaluating mRNA and protein expression at matched time points would help to clarify the relationship between transcriptional and protein-level changes. Supporting this interpretation, Jia et al. reported discrepancies between mRNA and protein expression levels in glioma cells cultured in 3D collagen scaffolds, suggesting that regulatory mechanisms beyond transcription may contribute to differences between gene and protein expression (54). Taken together, the present study suggests that our Gel: OPM:rGO scaffold microenvironment modulates cellular behavior through regulatory mechanisms that extend beyond transcriptional control, emphasizing the relevance of protein-level regulation in 3D tumor models. But, due to the limited number of independent biological replicates (*n* = 2), the Western blot findings should be considered preliminary.

The upregulation of mTOR, AKT, and PI3K in Gel: OPM:rGO and Matrigel cultures indicates altered expression of genes associated with the PI3K/AKT/mTOR signaling pathway, which is involved in regulating cell growth, proliferation, and survival. Similarly, the upregulation of Wnt3a and c-Myc indicates altered expression of genes associated with Wnt/β-catenin signaling, which plays an important role in cell proliferation and growth. Wnt3a is known to activates β-catenin signaling, leading to transcription of target genes such as c-Myc, which drives cell cycle progression and increases proliferative capacity (55–57).Elevated STAT3 and TNF-α expression may reflect alterations in genes associated with inflammatory and pro-survival signaling, which may contribute to a tumor-supportive microenvironment. In contrast, p53 expression remained unchanged across the 3D culture conditions. The unchanged p53 gene expression suggest that the Gel: OPM:rGO scaffold culture environment did not induce substantial changes in p53 transcription under the conditions examined. However, unchanged p53 mRNA levels do not necessarily indicate the absence of DNA damage-related stress, as p53 activity can also be regulated at the protein level and through post-translational modifications. Since p53 protein expression status was not assessed in the present study, the functional status of the p53 pathway cannot be fully determined based on transcript-level analysis alone. Future studies assessing p53 protein expression are needed to provide a more comprehensive understanding of p53 pathway activity under Gel: OPM:rGO scaffold culture conditions. However, as the present analysis was based primarily on mRNA expression, further protein-level and functional analyses are required to directly establish the activation status of the PI3K/AKT/mTOR, Wnt/β-catenin, and STAT3 signaling pathways.

Jia et al. reported that glioma cell lines (U87, U251, and HS683) cultured in 3D collagen scaffolds exhibited upregulated expression of genes associated with stemness, cell cycle regulation, apoptosis, EMT, migration, invasion, and overall glioma malignancy compared to 2D cultures (54). From this, it is evident that cells cultured in 3D systems exhibit altered or upregulated gene expression compared to 2D cultures (58). In line with these studies, our findings suggest that the 3D Gel: OPM:rGO scaffold culture system can markedly modulate gene expression, reflecting a physiologically relevant TME and exhibiting features that resemble those observed in Matrigel-based 3D culture.

Our findings demonstrated that cells cultured in Gel: OPM:rGO scaffold and Matrigel exhibited a glucose–lactate metabolic profile that more closely resembled the tumor phenotype when compared to 2D cultures, consistent with the Warburg effect. Specifically, our 3D culture system promoted enhanced glucose utilization accompanied by elevated lactate production, indicating a metabolic reprogramming toward aerobic glycolysis.

A similar observation was reported by Wang et al., who demonstrated that MCF-7 cells grown in collagen/alginate (CCA) fibrous scaffolds consumed significantly more glucose and released higher levels of lactate than cells maintained under 2D conditions, indicating a metabolic shift consistent with the Warburg effect (59). In line with this observation, our study showed differences in glucose and lactate levels in A549 cells cultured within the 3D scaffolds compared with 2D cultures. Taken together, these findings suggest that the 3D scaffold-based culture systems may influence the metabolic behavior of A549 cells and may provide a microenvironment associated with altered glucose and lactate metabolism compared with conventional 2D culture.

The enhanced cellular and TME-associated responses observed in the Gel: OPM:rGO scaffold compared with the Gel: OPM scaffold may be associated with the incorporation of rGO. The mechanisms by which rGO incorporation influences A549 cell behavior within the Gel: OPM:rGO scaffold are likely multifactorial. Our previous characterization demonstrated that incorporation of rGO improved the mechanical stability of the scaffold and enhanced its protein adsorption capacity, potentially through increased surface interactions. These physicochemical changes may influence cell–matrix interactions, cell attachment, and subsequent cellular behavior. In addition, the nanoscale surface characteristics of rGO may provide topographical cues that support cellular attachment and proliferation (22). A previous study by Rehman et al. also reported the favorable biocompatibility of rGO-containing biomaterials and their potential to enhance cellular proliferation, migration, and other biological responses (60).

In the present study, the Gel: OPM scaffold served as the rGO-free control, allowing the effects associated with rGO incorporation to be evaluated within the same scaffold system. Compared with the Gel: OPM scaffold, the Gel: OPM:rGO scaffold demonstrated enhanced cellular viability and tumor-associated responses, suggesting that rGO incorporation contributed to the development of a more favorable cellular microenvironment. Importantly, our previous biocompatibility assessment demonstrated high A549 cell viability in the Gel: OPM:rGO scaffold, with no apparent cytotoxic effects under the experimental conditions used (22). Together with the enhanced cellular viability observed in the present study, these findings suggest that rGO incorporation did not produce overt cytotoxic effects under the present culture conditions.

But, the observed differences cannot be attributed exclusively to a direct effect of rGO on A549 cell signaling, as the incorporation of rGO also modified the physicochemical and mechanical properties of the scaffold. Therefore, the enhanced TME-associated marker expression may result from indirect changes in the cell–matrix microenvironment rather than from a direct signaling effect of rGO alone. Furthermore, the present study did not directly determine whether the enhanced TME-associated marker expression in the Gel: OPM:rGO scaffold was primarily driven by rGO-associated mechanical cues, changes in surface chemistry, subtle ROS generation, electrical conductivity, or downstream signaling effects. Although no apparent cytotoxicity was observed, more subtle effects of rGO on intracellular signaling or redox regulation cannot be excluded. Further studies incorporating direct measurements of intracellular ROS, cell–matrix interactions, and relevant signaling pathways will therefore be required to clarify the specific mechanisms through which rGO modulates A549 cell behavior and TME-associated marker expression.

These results suggest that the Gel: OPM:rGO scaffold has potential as a 3D culture platform for modelling features of the lung TME. By supporting cellular, molecular, metabolic, and secretory responses associated with 3D tumor growth that showed similar overall trends to those observed in Matrigel, the scaffold demonstrates its potential to capture key characteristics of the 3D tumor milieu. The inclusion of the rGO-free Gel: OPM scaffold further demonstrated the contribution of rGO incorporation to the observed cellular responses. However, the present model is limited to an A549 monoculture and does not incorporate the diverse cellular components, such as fibroblasts, endothelial cells, macrophages, lymphocytes, and other stromal, that contribute to the multicellular complexity and heterogeneity of the native lung TME. Therefore, future studies incorporating more complex multicellular systems and patient-derived primary tumor cells will be necessary to further evaluate the ability of the scaffold to model TME interactions.

### Limitations and future studies

The limitation of the present study is that the practical applicability of the Gel: OPM:rGO scaffold for preclinical anticancer drug screening was not evaluated. Although the scaffold showed potential as a 3D culture model, its ability to assess chemotherapeutic responses remains to be established. Future studies assessing the responses of A549 cells cultured in the Gel: OPM:rGO scaffold to chemotherapeutic agents will be necessary to validate its suitability for preclinical drug screening. Another consideration of the study is that the Western blot analyses were performed using two independent biological replicates (*n* = 2) owing to limitations in experimental resources, whereas all other quantitative experiments were conducted using three independent biological replicates (*n* = 3). The limited sample size for the Western blot analyses may reduce the statistical robustness and reproducibility of the observed protein expression patterns. Therefore, conclusions derived from these analyses should be considered preliminary and interpreted with caution. Although the observed trends were consistent with the overall experimental findings, further validation using an increased number of independent biological replicates will be necessary to confirm the reproducibility and generalizability of these observations. Future studies will also focus on a comprehensive investigation of glucose–lactate metabolism and the Warburg effect to further elucidate the metabolic characteristics of A549 cells in the Gel: OPM:rGO culture systems.

## Conclusion

This study demonstrates the potential of the Gel: OPM:rGO scaffold as a three-dimensional culture platform for A549 lung cancer cells. The scaffold supported selected molecular and biochemical characteristics associated with the lung TME and exhibited overall patterns resembling those observed in Matrigel-based 3D culture. As the present system is based on an A549 monoculture, it provides a simplified model for investigating cellular, molecular, metabolic, and secretory responses and does not encompass the full multicellular complexity of the native lung TME. Nevertheless, the Gel: OPM:rGO scaffold may complement conventional 2D culture and existing Matrigel-based models as an *in vitro* platform for lung cancer research. Future research should focus on incorporating patient-derived tumor cells to further assess the physiological relevance and translational potential of the Gel: OPM:rGO scaffold.

## Data Availability

The original contributions presented in the study are included in the article/**Supplementary Material**. Further inquiries can be directed to the corresponding author.
