# Supplementary material for: Reduced graphene oxide-incorporated gelatin-oxidized polymannose scaffold-based 3D Lung cancer model for mimicking the tumor microenvironment
Source: Front Oncol. 2026 Sep 24;16:1955340. doi: 10.3389/fonc.2026.1955340 (PMC13645567; doi:10.3389/fonc.2026.1955340)
Supplement: Supplementary file 1 [file SupplementaryFile1.docx]

**Reduced graphene oxide-incorporated gelatin-oxidized polymannose scaffold-based 3D Lung cancer model for mimicking tumor microenvironment**

*Hema Priya Manivannan^1^, Vishnu Priya Veeraraghavan^1^, K. P. Ameya^2^, Durairaj Sekar^2^, Arul Prakash Francis^1^**

^1^Centre of Molecular Medicine and Diagnostics (COMManD), Department of Biochemistry, Saveetha Dental College and Hospitals, Saveetha Institute of Medical and Technical Sciences, Saveetha University, Chennai-600077, India.

^2^RNA Biology Lab, Saveetha Dental College and Hospital, Saveetha Institute of Medical and Technical Sciences, Saveetha University, Chennai-600077, India.

***Corresponding Author**

Email: fdapharma@gmail.com; arulprakashf.sdc@saveetha.com

ORCID ID: https://orcid.org/0000-0003-1357-6466

**Funding**: The authors acknowledge the financial support for the Start-up Research Grant (SRG/2022/001432) by the Anusandhan National Research Foundation, Department of Science and Technology, Government of India, New Delhi, India.

**Supplementary Note:** The detailed fabrication procedures, physicochemical characterization, and biocompatibility of the Gel:OPM and Gel:OPM:rGO scaffolds were previously reported in our earlier study (1). For ease of reference and to provide the relevant biomaterial context for the present study, the key scaffold properties relevant to the observed cellular responses are summarized below.

**Summary of Previously Reported Scaffold Fabrication and Characterization**

Briefly, 3% gelatin and 5% oxidized OPM were individually dissolved in borate buffer. rGO at a concentration of 0.01% was added to the OPM solution and sonicated to ensure uniform dispersion. For scaffold formation, 0.25 mL each of the gelatin and rGO-incorporated OPM solutions was dispensed into individual wells of a 24-well plate, mixed thoroughly, and allowed to undergo gelation at room temperature for 10 minutes. The combination of OPM and gelatin resulted in an in situ self-gelling hydrogel via a single-step process. Following gelation, the plates were frozen at −20 °C and then transferred to −80 °C overnight. The frozen samples were subsequently lyophilized for 48 hours to obtain the final Gel:OPM:rGO scaffolds, which were then used for further analyses. In parallel, Gel: OPM hydrogels without rGO were fabricated using the same procedure and served as the control for comparative evaluation.

**Swelling and degradation behaviour:** The swelling and degradation behaviour of the scaffolds was evaluated by incubating them in phosphate-buffered saline (PBS) for the specified time periods. Both Gel:OPM:rGO and Gel:OPM scaffolds exhibited rapid swelling during the initial period (0–30 min), followed by no significant changes, reaching equilibrium swelling within 60 min. This behaviour was attributed to the hydrophilic nature and porous network of the scaffolds, which facilitated rapid water absorption. Among the scaffolds, Gel:OPM:rGO exhibited the highest equilibrium swelling ratio. Regarding degradation, the 3:5 Gel:OPM:rGO scaffold showed delayed degradation, occurring at approximately 15 days, whereas the Gel:OPM scaffold degraded earlier. The delayed degradation of the Gel:OPM:rGO scaffold was attributed to the incorporation of rGO, which enhanced scaffold stability and prolonged its degradation time. These properties indicate that the Gel:OPM:rGO scaffold provides suitable swelling and degradation characteristics for longer-term cell culture.

**Porosity:** Scaffold porosity was determined using the solvent replacement technique by recording the dry weight before and after overnight immersion in ethanol. Both the 3:5 Gel:OPM and 3:5 Gel:OPM:rGO scaffolds exhibited a porous structure. The increased porosity associated with the lower gelatin ratio suggests that reducing the gelatin content promotes the formation of a more open scaffold network, which may facilitate solvent flow.

**Compression testing:** The compressive mechanical properties of the scaffolds were evaluated using a universal testing machine (Instron). The Gel:OPM:rGO scaffold exhibited higher compressive stress (0.05 ± 0.01 MPa) and a slightly higher strain at break (8.78 ± 0.93%) compared with the Gel:OPM scaffold (0.02 ± 0.00 MPa and 7.51 ± 2.49%, respectively), indicating that rGO incorporation improved the mechanical strength and stability of the scaffold.

**Biocompatibility:** The biocompatibility of the Gel:OPM:rGO and Gel: OPM scaffolds was evaluated using an Alamar Blue assay following the culture of A549 cells on the scaffolds for 2, 4, and 7 days. Both scaffolds supported A549 cell viability and proliferation over the culture period, with the Gel:OPM:rGO scaffold showing higher cell viability and metabolic activity than the Gel:OPM scaffold. The increased cell viability observed over time, particularly on day 7, indicates that the Gel:OPM:rGO scaffold provides a supportive environment for A549 cell growth.

**Supplementary Table S1.** Primer Sequences for TME genes

| **Gene** | **Sequence** | **Function** |
| --- | --- | --- |
| VEGF-A -Forward | AGGGCAGAATCATCACGAAGT |  |
| VEGF-A- Reverse | AGGGTCTCGATTGGATGGCA |  |
| MMP9- Forward | CCTGGAGACCTGAGAACCAATC | Extracellular matrix (ECM) degradation marker |
| MMP9- Reverse | GATTTCGACTCTCCACGCATCT |  |
| VIM- Forward | GAGAACTTTGCCGTTGAAGC | Epithelial-Mesenchymal Transition (EMT) marker |
| VIM- Reverse | GCTTCCTGTAGGTGGCAATC |  |
| ALK Forward | CGACCATCATGACCGACTACAA |  |
| ALK Reverse | CCCGAATGAGGGTGATGTTTT |  |
| EGFR-Forward | TTTGGGAGTTGATGACCTTTGG | Growth factor Receptor marker |
| EGFR-Reverse | ACGGAACTTTGGGCGACTATCT |  |
| GAPDH- Forward | GTGAAGGTCGGAGTCAACG | Housekeeping gene |
| GAPDH- Reverse | TGAGGTCAATGAAGGGGTC |  |

**Supplementary Table S2.** Primer Sequences for pathway genes

| **Gene** | **Sequence** |
| --- | --- |
| MTOR - Forward | CGCTGTCATCCCTTTATCG |
| MTOR- Reverse | ATGCTCAAACACCTCCACC |
| AKT1 - Forward | GGAGGACAATGACTACGGC |
| AKT1- Reverse | TCATCTTGGTCAGGTGGTGT |
| PIK3CA - Forward | GGTTGTCTGTCAATCGGTGACTGT |
| PIK3CA - Reverse | GAACTGCAGTGCACCTTTCAAGC |
| wnt3a - Forward | TCCACGCCATTGCCTCAG |
| wnt3a - Reverse | CGAGACACCATCCCACCAAA |
| STAT3 - Forward | CTTTGAGACCGAGGTGTATCACC |
| STAT3 - Reverse | GGTCAGCATGTTGTACCACAGG |
| CMYC - Forward | TACCCTCTCAACGACAGCAG |
| CMYC - Reverse | TCTTGACATTCTCCTCGGTG |
| TNFα - Forward | ATGAGCACTGAAAGCATGATC |
| TNFα - Reverse | AAAGTGCAGCAGGCAGAAGA |
| P53 - Forward | TCAGTCTACCTCCCGCCATA |
| P53 - Reverse | TTACATCTCCCAAACATCCCT |

**Supplementary Figure S1**. Heatmap illustrating the TME gene expression in (a) Gel:OPM:rGO (b) Gel:OPM, (c) Matrigel Culture relative to 2D.

**Supplementary Figure S2**. Heatmap illustrating the signaling pathway gene expression in (a) Gel:OPM:rGO (b) Gel:OPM, (c) Matrigel Culture relative to 2D.

**Reference**

(1). [Manivannan HP, Veeraraghavan VP, Francis AP. Development of reduced graphene oxide-incorporated gelatin-polymannose hydrogel scaffolds as a 3D cell culture model for cancer research. *J Biomater Sci Polym Ed* (2026)1–38.](http://paperpile.com/b/gik6Pm/INp1D)

**Western Blotting - Orginal**

**VIM**

**VEGF A**

**MMP 9**

**GAPDH**

**EGFR**

**ALK**

**Western Blotting - Duplicate**

**GAPDH**

**EGFR**

**VIM**

**MMP 9**

**VEGF A**

**ALK**
